# Sexing of Adults for Three Mexican Pine Cone Beetle Species, *Conophthorus conicolens*, *C. teocotum*, and *C. michoacanae*

**DOI:** 10.1673/031.011.17001

**Published:** 2011-12-12

**Authors:** Adolfo Del Rio Mora, Oksana Tuchina

**Affiliations:** ^1^ Instituto de Investigaciones Agropecuarias y Forestales, Universidad Michoacana de San Nicolas de Hidalgo, Morelia, Michoacán, México; ^2^School of Engineering and Science, Jacobs University, Campus Ring 6, Research II, Room 38, D-28759 Bremen, Germany

**Keywords:** setae

## Abstract

Individuals belonging to species of the genus *Conophthorus* (Coleoptera: Curculionidae) are notoriously difficult to sex, especially if only certain parts of the beetle are available and the genitals are unidentifiable. We are presenting evidence demonstrating that rows of setae and setal characteristics at the posterior margin of tergite VII (the fifth visible tergite) can be used to sex the three Mexican pine cone beetle species *Conophthorus conicolens* Wood, *C. teocotum* Hopkins, and *C. michoacanae* Wood, from each other. Although the three *Conophthorus* are clearly described taxonomically by Wood (1977), the continued increase and development of forest plantations with a mixture of host species elucidates the importance of having reliable methods of sexing these species for purposes of researching and monitoring programs of their populations and pest management and control.

## Introduction

Pine cone beetles of the genus *Conophthorus* (Coleoptera: Curculionidae) are destructive pests of pine trees in North America (Hedlin et al. 1980), and each year they damage ∼15-60% of the crop in Mexico alone (Del Rio and Mayo 1988). Guillete and co-workers (Rappaport et al. 2000) reported how different behavioral and semiochemical methods could be used to monitor the numbers of beetles in Mexican forests, and later Del Rio et al. (2007) discovered a way to prevent damage to the pine cones by spraying protective semiochemicals.

The three *Conophthorus* species studied, *C. conicolens* Wood, *C. teocotum* Hopkins, and *C. michoacanae* Wood are specialists feeding only on cones of hard pine species (Diploxilon). One of them, *C. conicolens* has several hosts; *Pinus leiophylla*, *P. douglasiana, P. lawsoni, P. montezumae,* and *P. pseudostrobus.* Thus, this species is abundant and widely distributed in the Western Sierra and forests in the central region of Mexico along with populations of *C. ponderosae,* which feeds on the same hosts. *Conophthorus teocotum* and *C. michoacanae* have unique hosts; cones of *P. teocote* and *P. michoacana,* respectively.

In order to investigate new methods of protecting pines it is important to know as many behavioral, anatomical, and physiological details of the beetles as possible (Campbell and Borden 2006). Additionally, a reliable method of sexing *Conophthorus* individuals is necessary, as sometimes the genitalia, for whatever reason, are not identifiable, or only fragments of a beetle remain, such is the case in the feeding debris of spider webs.

Although most Mexican pine beetles have not been studied intensively until now, and only the biology of *C. conicolens* has been reasonably well documented by Del Rio and Mayo (1988), Herdy (1959) pointed out that abdominal tergite morphology may be used to distinguish males from females. Santiago-Blay and Young (1995) studied two *Conophthorus* species (*C. coniperda* and *C. ponderosae*) and found that male beetles have a row of bifid or trifid setae at the posterior margin of tergite VII (the fifth visible tergite), which can be seen under the light microscope at magnification 40×. The present work was done in order to study whether the sex of other *Conophthorus* species can also be distinguished by the morphology of the posterior margin of tergite VII (the fifth visible tergite). This study investigated specimens of *C. conicolens, C. teocotum,* and *C. michoacanae,* which damage *P. pseudostrobus, P. teocote,* and *P. michoacanae,* respectively.

## Materials and Methods

Three species of beetles (*C. conicolens, C. teocotum,* and *C. michoacanae*) were collected in the Michoacan pine Forests (Mexico) during summer time. The cones with adult insects inside were carefully dissected, and beetles of both sexes were extracted, dehydrated, and cleaned in a graded series of ethanol (50, 70, 90 and 2 × 100 % for 10 min each). The tergites of fifteen pairs of male and female individuals of three species were prepared and assembled for comparison and study; samples were then air dried and coated with gold by an EMI Tech K550X sputter coater (Quorum Technologies Inc, www.quorumtechnologies.com) for 2–3 min to a thickness of approximately 20 nm. Examinations took place at Jacobs University Bremen, Germany, under a JEOL JSM-5900 (JEOL Ltd, www.jeol.com) scanning electron microscope operated at a voltage of 15–20 kV.

**Figure 1.**
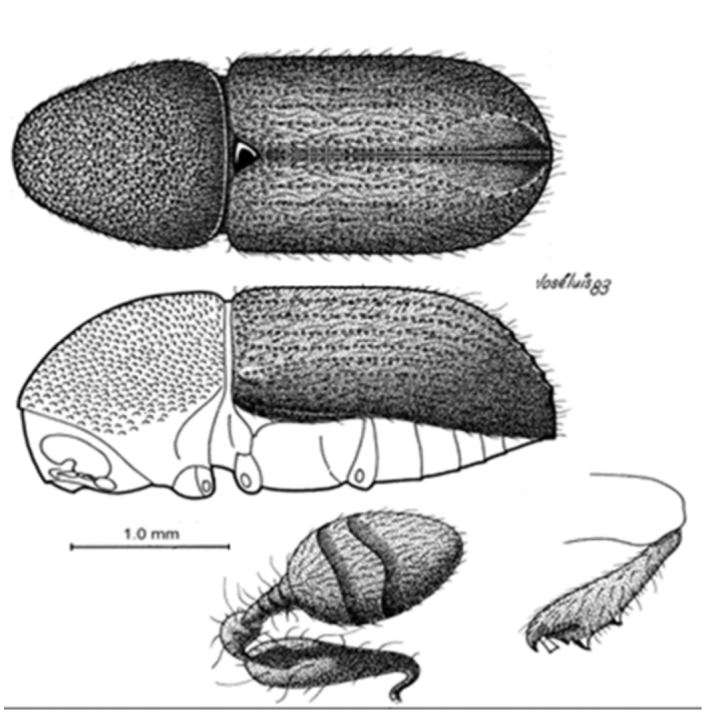
*Conophthorus conicolens*: Dorsal and lateral view, club, and foreleg section. High quality figures are available online.

## Results and Discussion

**Figure 2.**
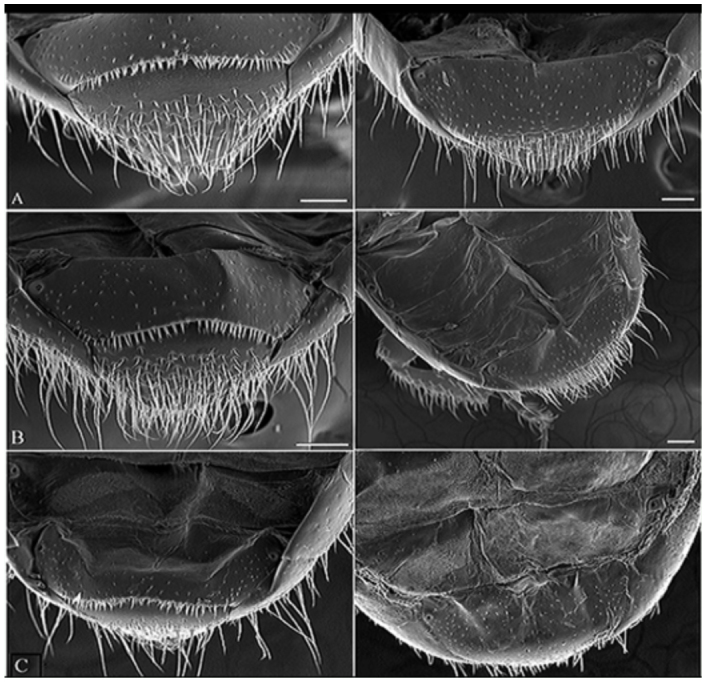
Scanning electron micrographs of male (left side) and female (right side) abdominal tergites in three pine beetles: (A) *Conophthorus conicolens,* (B) *Conophthorus teocotum* and (C) *Conophthorus michoacanae.* Scale bar = 100 µm. High quality figures are available online.

The female individuals of all three species did not possess any setae on the fifth visible tergite; the surface of the latter is flat and its edge is smooth, but in the males of all three species setae are developed in these places. The setae at the posterior edge of the fifth visible tergite in *C. conicolens* and *C. michoacanae* males are bifid or trifid and shorter than those seen in male *C. teocotum* specimens (Figure 2). In *C. conicolens* and *C. michoacanae* the setae are about 19 µm long and 8 µm wide at their base and therefore closely resemble those of male *C. ponderosae,* described by Santiago-Blay and Young (1995). However, in male *C. teocotum,* the setae measured up to 27 µm in length and 8 µm in width at the base and are long and slender and mostly bifid.

**Figure 3.**
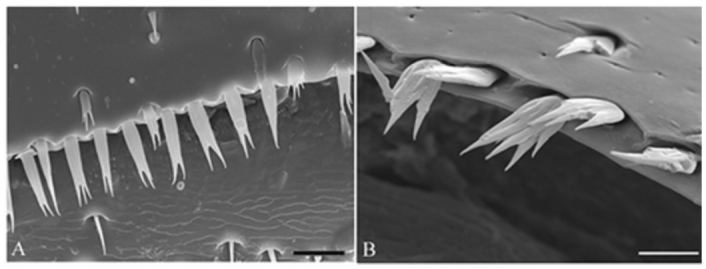
Rows of setae on the posterior margin of tergite VII (fifth visible tergite) in males of (A) *Conophthorus teocotum* and (B) *Conophthorus conicolens.* Note different magnification for (A) and (B): scale bar = 20 µm in (A) and 10 µm in (B). High quality figures are available online.

The described character should facilitate sexing of individuals belonging to the three species in question and more easily allow gender-specific behavioral and physiological experiments to be devised.

These cone borer species vary in size, but for *C. conicolens* and *C. teocotum,* most male and female specimens measured an average of 3.5 mm in length, while *C. michoacanae* measured 4.2 mm.
